# Reviews

**Published:** 1879-11

**Authors:** 


### Reviews.

**Treatise on Hygiene and Public Health.** Edited by Albert H. Buck, M. D., American editor of Ziemsen's Cyclopedia of the Practice of Medicine, Instructor in Otology in the College of Physicians and Surgeons in New York. New York: Wm. Wood & Co.

This work is in two large volumes of 792 and 657 pages respectively. The first volume is made up of a series of articles written by men deservedly eminent in the medical profession, and is divided into two parts, the first being devoted to the subject of Individual Hygiene, part second to Habitations. The volume opens with a concise and thoughtful article from the pen of Dr. Billings, Vice-President of the National Board of Health, in which, after some prefatory remarks, he dwells upon the Causes of Disease and the Jurisprudence of Hygiene. This is followed by a series of five papers, of which space will allow us to give their titles only, viz.: Infant Hygiene, by Dr. Jacobi, Professor of Diseases of Children, College of Physicians and Surgeons, New York. Food and Drink, by Dr. James Tyson, Professor of General Pathology and Morbid Anatomy in the University of Pennsylvania. Drinking Water and Public Water Supplies, by Professor W. R. Nichols, of Massachusetts Institute of Technology. Physical Exercise, by Dr. A. B. Ball, of New York. The Care of the Person, by Arthur Van Harlingen, Chief of the Clinic for Diseases of the Skin, Hospital of the University of Pennsylvania.

The articles in part II are: first, a paper on Soil and Water, by Dr. W. H. Ford, President of the Board of Health, Philadelphia, Pa.; second, an article on *The Atmosphere*, by D. F. Lincoln, M. D., of Boston, in which the subject is studied under the four heads of: I, Natural Components of Air; II, Impurities; III, Meteorology and Climate; and IV, Ventilation and Heating. The volume closing with an article, written by Dr. Francis H. Brown, of Boston, on the General Principles of Hospital Construction. These papers are very instructive and valuable, and should be read and studied not only by Physicians and Sanitarians, but by all educated persons.

Volume II is also divided into two parts, under the general titles of Occupation and Public Health. Dr. Roger S. Tracy, Sanitary Inspector of the Board of Health, New York, contributes the first article on The Hygiene of Occupation, and this excellent paper is followed by the following, all equally well written:

The Hygiene of Camps, by Charles Smart, M. B. C. M., Assistant Surgeon, U. S. Army.

The Hygiene of the Navy and Merchant Marine, by Thos. I. Turner, M. D., Medical Director, U. S. Navy.

The Hygiene of Coal Mines, by Henry C. Shaefer, Coal editor of the *Miners' Journal*, Pottsville, Pa.

The Hygiene of Metal Mines, by R. W. Raymond, Ph. D., editor of the *Engineering and Mining Journal*, New York City.

The second part of volume II opens with valuable articles on Infant Mortality and Vital Statistics, by Thos. B. Curtis, M. D., Surgeon to out patients, Massachusetts General Hospital.

The well-known chemist, S. P. Sharples, Inspector of Milk for the City of Cambridge, contributes a first-rate article on the Adulteration of Food.

The fourth article is on Public Nuisances, and was written by Roger S. Tracy, M. D., Sanitary Inspector of the Board of Health, New York.

Then follows Quarantine, with reference solely to seaport towns, by S. O. Vanderpoel, M. D., Health Officer of the port of New York.

Inland Quarantine, by S. S. Herrick, Secretary of the Louisiana State Board of Health.

Small pox and other contagious diseases (scarlet fever, measles, whooping cough), written by Allen McLane Hamilton, M. D., Sanitary Inspector of the Board of Health, New York, and Bache Emmett, M. D., New York City.

The Hygiene of Syphilis, by F. R. Sturges, M. D., Clinical Lecturer on Venereal Diseases, in the University of New York, etc.

Disinfectants, by Elwyn Waller, Ph. D., Chemist to the Metropolitan Board of Health, New York.

Village Sanitary Association, by Roger S. Tracy, Sanitary Inspector of the Board of Health, New York.

An article on the important subject of School Hygiene is well written by Dr. D. F. Lancaster, of Boston, and this closes a work the great value of which will be immediately recognized by scientific physicians, and could it enter the house of every man of education, and there receive the study and attention which it deserves, the work would be of incalculable benefit. In the crowded state of our pages, and especially in this department of book reviews, we can not, in justice to ourselves, or the authors, attempt a review of the separate papers, but every one of them is worthy of study. Some of them are very elaborate, and all are thoughtful and suggestive on topics relative to the health and well-being of the community. As a whole, the book can receive nothing but favorable criticism. It contains a fund of useful and trustworthy facts and conclusions, which possess permanent value. Every subscriber to Ziemssen should complete that great Cyclopædia by obtaining this work, and every educated man should buy this first really comprehensive treatise on private and public Hygiene, written with special reference to the different climates, conditions of soil, habitations, modes of life and laws of the United States. Messrs. Wood & Co. are entitled to much credit for the admirable dress in which the work is presented. The type is large and the paper excellent, and illustrations have been freely introduced. D.

---

**"Eye Sight and How to Care for It."**

This is a very large name for a very small book. The author, Dr. Harlan, presents the subject in a popular manner; however, the illustrations are good, and altogether this volume of the "American Health Primers" must prove as acceptable to the public, and as profitable to Lindsay & Blakiston, as are the others of the series. H.

---

**The National Dispensatory.** Containing the Natural History, Chemistry, Pharmacy, Actions and Uses of Medicines; including those recognized in the Pharmacopœia of the United States, Great Britain, and Germany. With numerous references to the French Codex. By ALFRED STILLE, M. D., LL. D., Prof. Theory and Practice of Medicine, and of Clinical Medicine, in the University of Pennsylvania, and JOHN M. MAISCH, Pharmaceutical Doctor, Prof. of Materia Medica, in the Philadelphia College of Pharmacy. Second Edition, thoroughly revised, with numerous additions. Philadelphia: Henry C. Lea.

The first edition of this great work appeared only a few months ago, and that the publishers should find it necessary to issue a second edition in the same year, is most conclusive evidence that this work has really supplied a want felt by the medical and pharmaceutical profession. The authors have taken advantage of this opportunity for revision, and though, in the short time that has elasped since the first edition appeared, nothing really new or important has been brought to light, still some valuable additions and alterations have been made, and the errors and imperfections in the first edition, which it is only justice to say were marvellously few for so large a work, have been corrected.

The book is now a handsome octavo volume, of 1672 closely printed pages, with 239 illustrations. The material embodied in the work is truly immense, as shown by the almost countless number of subjects treated. It is now, undoubtedly, the most perfect book of its kind in any language. D.

---

**Analysis of the Urine, with special reference to the Diseases of the Genito-Urinary Organs.** By K. B HOFFMANN, Professor in the University of Gratz, and R. ULTZMANN, Docent in the University of Vienna. Translated by T. B. BRUNE, M. A., M. D., and H. H. CURTIS, Ph. B. New York: D. Appleton & Co. Buffalo: Ulbrich & Kingsley.

This work enjoys a well-deserved popularity in Germany and Austria, and already it has been translated into three languages.

That it is likely to be appreciated in America, is shown by the fact that two translations of it have appeared here.

In a recent number of the Journal, we reviewed the book as translated by Dr. Forchirmer, and published by a Cincinnati house. The work now on our table is published by D. Appleton & Co., and its appearance is worthy of their reputation. It is printed in large clear type, on excellent paper, and the book is made doubly valuable to the student by the admirable plates. These do not appear in the German edition, but have been taken from photographs furnished by Dr. Ultzmann, and from the author's "Atlas der Physiologischen und Pathologischen Harnsedimente." In noticing Dr. Forchirmer's translations, we spoke highly of the work, and again we commend it to the attention of the student and general practitioner, and we would advise our friends to secure the work with the Appleton imprint. D.

---

**A Guide to Surgical Diagnosis**. By CHRISTOPHER HEATH, F. R. C. S., Surgeon to University College Hospital, etc. Philadelphia: Lindsay & Blakiston.

The object of this little book is to assist the student of surgery in forming a diagnosis of cases coming before him, and it will without doubt be a useful book for the student to have in his pocket during his clinical lectures and studies in the hospital. The book is arranged in twenty-five chapters, each of which treats of the diagnosis of diseases occurring in one of the surgical regions. No attempt is made to discuss the pathology or the treatment of any of the disorders described. The book contains many tables, for instance, about differential diagnosis of inguinal tumors, femoral tumors, scrotal tumors, etc., by which arrangement the usefulness of the book can but be increased. M.

---

**The Advantages and Accidents of Artificial Anæsthesia**. By LAWRENCE TURNBULL, M. D. Second edition Philadelphia: Lindsay & Blakiston.

This monograph cannot fail to be of unusual interest to every intelligent practitioner, in spite of all that has been written on the subject. The principal anæsthetic agents are taken up in order, and the important features of each carefully discussed. The various indications for using or avoiding them, together with the manner and probable cause of death in unfortunate cases, are pointed out in detail. The book is written in the same clear style which pervades the "Diseases of the Ear," by the same author, and if a physician were to read only the chapter on ether and chloroform, he would find himself well satisfied with his bargain. H.

---

**Modern Surgical Therapeutics.** A compendium of current formulæ, approved dressings and specific methods for the treatment of surgical diseases and injuries. By GEORGE H. NAPHEYS, A. M , M. D., etc. Sixth edition. Revised to the most recent date. Philadelphia: D G. Brinton. 1879.

It is but a few months since we called the attention of our readers to Dr. Napheys' Medical and Surgical Therapeutics, works which go hand in hand with each other, and together comprise the present therapeutics of both medicine and surgery. The sixth edition has been enlarged and improved, and will add to the value and great popularity of the work. J. F. M.

---

**American Health Primer.** The Summer and its Diseases. By JAMES D. WILSON, M. D. Philadelphia: Lindsay & Blakiston. 1879. Buffalo: Butler.

The contents of this little work are comprised in seven chapters, of which the first treats of summer; second, sunstroke and heat fever; third, summer diarrhœa and dysentery; fourth, cholera infantum; fifth, summer and autumnal fevers; sixth, summer colds and hay asthma; seventh, the skin and its diseases.

A great amount of valuable information for the prevention, as well as cure of disease, is condensed in its pages, which will be of great benefit to the general reader. The general practitioner also who desires a small work, elucidating general principles of sanitary science, will find in this work a useful guide. L.

**On Loss of Weight, Blood Spitting and Lung Disease.** By HORACE DOBELL, M. D., etc., etc., consulting Physician to the Royal Hospital for diseases of the chest, late Senior Physician to the Hospital, etc , etc. Philadelphia: Lindsay & Blakiston.

The author discusses, with great care and earnestness, the questions of what is the import of loss of flesh and loss of blood, or hæmoptysis as prodromata of tuberculosis or consumption. He says, "the interdependence of loss of weight, blood spitting and lung disease is so intricate that, although either may occur without the others, neither can be properly treated without considering the rest." The discussion on these questions is comprehensive and complete. Eight hundred cases, as they occurred in the Royal Hospital, are reported, and a chart showing the relation between loss of weight, coughs, blood spitting and lung disease in one hundred male cases of hæmoptysis accompanies the work, and adds to the force of the text. J. F. M.

---

**The Heart and its Diseases; with their treatment, including the Gouty Heart.** By J. MILNER FOTHERGILL, M. D., Member of the Royal College of Physicians of London, etc. Second edition. (Entirely re-written.) With illustrations. Philadelphia: Lindsay & Blakiston. 1879. 476 pp.

The author in his preface claims that while the literature on heart disease "contains the systematic works of Hope, Stokes, Walsh, and our American confrère Flint, scientific progress may have made room for a newer treatise." Acting upon this belief, he essays to add another to the many important works already published on cardiac diseases, and we think the task has been successfully accomplished. The present work constitutes a really valuable treatise, which will be cordially received by the profession, and be regarded as an important addition to our literature on cardiac disease. There are many features in this work which are especially commendable. The author strives to describe each form of disease of the heart, not as a combination of signs and symptoms, but as possessing a natural history.

Indications for treatment are thus furnished, which are based on philosophical principles. The author pays a merited tribute of praise to his German and English co-laborers, and recognizes the rapid progress made in placing cardiac pathology and therapeutics on a sound scientific basis. L.

---

**The Student's Guide to the Diseases of Women.** By ALFRED LEWIS GALABIN. M. A., M. D., F. R. C. P. With sixty-three illustrations. Philadelphia: Lindsay & Blakiston. 1879. Buffalo: Butler.

This work aims to present to the student of medicine, within the limits of a small volume of 370 pages, a condensed account of the diseases peculiar to women. We object, as a rule, to a treatise upon so broad a subject as Gynecology, which can scarcely touch upon the salient points of the pathological conditions it endeavors to elucidate. The author has, however, succeeded in simplifying many subjects, and by aid of illustrations shows the practical method of performing many important minor operations. But he treats so briefly of many diseases, that his conciseness robs several chapters, devoted to very important subjects, of the clearness of description so essential to the student for whose especial benefit the work is written. At the same time the work contains much to commend. Of its kind, it is the best we have read, and if carefully studied, as an introduction to this important department, to be supplemented by a more thorough examination and study of the larger works of Barnes, Byford, Thomas and Emmet, recognized all over the world as authority in this field of professional labor, it will subserve a very useful purpose in guiding the student to sources of knowledge, from which, for a time, he may chance to be denied access.

